# MRCPsych Course – a National Perspective

**DOI:** 10.1192/bjo.2026.11314

**Published:** 2026-06-30

**Authors:** Joanne Hew, Fergus Lewis

**Affiliations:** 1 North East London NHS Foundation Trust, London, United Kingdom; 2 South London and Maudsley NHS Foundation Trust, London, United Kingdom

## Abstract

**Aims:**

To gather the views of UK-based resident doctors on their local MRCPsych courses.

**Methods:**

MRCPsych courses aim to broaden resident doctors’ knowledge of psychiatry andprepare them for the Royal College of Psychiatrists membership examinations. The courses vary widely between deaneries. Understanding resident doctors’ experiences is needed to help ensure equitable and high-quality delivery.

A cross-sectional survey was created and disseminated to UK-based resident doctors via the Psychiatric Resident Doctors Committee. Information was gathered on satisfaction, barriers and suggestions for improvement.

**Results:**

A total of 25 responses were received.

69% of respondents felt that their MRCPsych course is useful for their development as psychiatrists, but only 36% felt it was helpful in preparing them for the examinations.

Respondents valued interactive sessions, online flexible learning and recorded sessions, practical sessions, and found it helpful when sessions followed the syllabus. They preferred when sessions were delivered by a clinician and highlighted teaching on subspecialties as particularly helpful due to lack of exposure in certain regions.

Respondents found their courses less helpful when the sessions did not follow the syllabus, out of date material was used, and when there was no introduction to the MRCPsych examinations. Respondents preferred if lecturers had a knowledge of their syllabus and format of examinations. They also highlighted issues with travel when courses were held far away from their base.

Respondents suggested improvements could be made by having a set curriculum and structure, addition of simulation-based learning, more CASC teaching and incorporating lived experience into the sessions. They preferred to be informed of the topic in advance so they could engage in pre-learning activities. They suggested that co-production of the course with themselves and relevant stakeholders would result in a higher quality and more useful course.

79% of respondents reported that they get protected time for attending the course, with 17% maybe and 4% no. Common issues cited were on-call requirements and people working on inpatient wards finding it difficult to leave due to higher demands.

Respondents identified using online paid-for resources and question banks, reading textbooks and guidelines, small group practice and attending conferences as supplementary activities to support their learning.

**Conclusion:**

The MRCPsych course is well-received and appreciated by resident doctors, however there were some suggestions about how it could be improved. A more standardised approach could be helpful to ensure equitable access to high quality courses across the UK.

